# The Role of Neuro‐Immune Regulation in the Pathogenesis of Psoriasis

**DOI:** 10.1155/jimr/3016265

**Published:** 2026-02-09

**Authors:** Xin Liu, Mingyu Yu, Pan Zou, Suwen Liu, Jian Jiang, Liyang Ge, Su Jiang, Jingyu Zhang, Xin Wang, Hongxiang Chen

**Affiliations:** ^1^ Department of Dermatology, Zhongnan Hospital of Wuhan University, No. 169 Donghu Road, Wuchang District, Wuhan, 430071, Hubei, China, znhospital.cn; ^2^ Department of Dermatology, Union Hospital, Tongji Medical College, Huazhong University of Science and Technology, Wuhan, 430022, China, hust.edu.cn

**Keywords:** immunity, inflammatory response, neuropeptides, psoriasis, sensory neurons

## Abstract

Psoriasis is a chronic inflammatory skin disease characterized by abnormal proliferation of epidermal cells induced by an overactive immune system. In addition to the interaction between immune cells and keratinocytes, emerging research highlights the indispensable role of peripheral sensory neurons in the initiation and progression of psoriatic inflammation. Sensory neurons not only perceive various external stimuli but also participate in skin immune regulation and barrier repair through the release of neuropeptides and neurotransmitters. This review systematically compiles current research advancements concerning the altered neural innervation patterns in psoriatic lesions, the pathophysiological functions of sensory neuron‐specific receptors and ion channels, and the regulatory mechanisms of neuropeptides in disease pathogenesis. By elucidating the precise contributions of sensory neurons to the development of psoriasis, this work seeks to further clarify their specific role in the disease and provide new insights for enhancing the understanding of its pathogenesis, thereby informing the development of more targeted therapeutic strategies.

## 1. Introduction

Psoriasis is a common chronic inflammatory skin disease, characterized by erythematous, scaly plaques, primarily affecting the scalp, knees, elbows, and lower back. Globally, approximately 125 million people suffer from psoriasis, which not only significantly impairs physical health but also imposes a substantial psychological burden on patients [1]. The pathogenesis and triggers of psoriasis are complex and not yet fully understood. Recent retrospective analyses of anti‐PD‐L1 [2] and pembrolizumab [3]‐induced psoriasis have further highlighted the intricate immune dysregulation involved in its onset. Nonetheless, clinical observations have reported spontaneous resolution of psoriatic lesions in certain patients following injury to the central or peripheral nervous systems, suggesting a potential regulatory role of neural innervation in the modulation of psoriatic inflammation [4]. Furthermore, numerous studies have corroborated that psychological factors, such as insomnia, depression, and anxiety, substantially exacerbate psoriatic lesions, underscoring the significance of neurogenic factors in disease progression [5–7]. These findings imply that neural regulation may be a critical factor in the advancement of psoriatic lesions.

The skin, as a sensory organ richly innervated by sensory nerve fibers, plays a crucial role in the body’s perception and defense mechanisms [8]. These sensory nerve fibers respond to diverse noxious stimuli, such as mechanical, thermal, and chemical inputs, and are consequently termed nociceptive sensory neurons [9]. Upon activation by such stimuli, these neurons generate action potentials to convey signals to the central nervous system [10]. Concurrently, nociceptive sensory neurons can release a variety of neuropeptides that modulate local immune responses, thereby playing a pivotal role in the skin’s immune defense and inflammatory processes [11]. Traditionally, the nervous and immune systems have been examined as separate entities. However, accumulating evidence reveals complex bidirectional interactions between these systems in various organs, including the skin [12, 13]. The skin is richly innervated by primary sensory neurons, which allow it to detect a range of external stimuli. Upon encountering potential threats, the sensory nervous system rapidly initiates nociceptive signaling, including pain and pruritus, to alert the organism to imminent danger, which prompts actions like withdrawal reflexes and scratching behaviors to mitigate damage and preserve bodily integrity [14].

Sensory neurons that innervate the skin are characterized as pseudo‐unipolar, possessing one axonal branch that terminates in the spinal dorsal horn and another that extends to the peripheral skin. The cell bodies of these neurons are situated in peripheral ganglia. Specifically, those innervating the face and oral cavity are located in the trigeminal ganglion, while those serving the remainder of the body are found in the dorsal root ganglia (DRGs) [15, 16]. Sensory nerve terminals express various channels, including transient receptor potential (TRP) channels, purinergic 2X receptors (P2XRs), mechanosensitive ion channels and G protein‐coupled receptors (GPCRs). Upon exposure to itch or pain stimuli, cation channels such as TRPV1 or TRPA1 on sensory nerve terminals become activated, resulting in membrane depolarization and the subsequent activation of voltage‐gated sodium channels such as NaV1.7 and NaV1.8. This process transmits itch signals to the spinal dorsal horn and subsequently to the brain for perception [15, 16].

Additionally, sensory nerve terminals also express various inflammatory cytokine receptors, which respond to immune cell‐derived inflammatory mediators, thereby modulating pruriceptive and nociceptive signaling in cutaneous inflammatory pathologies [17, 18]. For instance, interleukin‐4 (IL‐4), a type 2 inflammatory cytokine primarily secreted by Th2 cells, plays a significant role in atopic dermatitis (AD) [19]. The absence of IL‐4 receptors (IL‐4Rs) on sensory neurons significantly alleviates scratching behavior in AD mice, indicating the importance of IL‐4R‐mediated signaling in AD‐related itch [20]. Moreover, IL‐4R activation on sensory neurons enhances itch responses induced by histamine, chloroquine, and thymic stromal lymphopoietin (TSLP) [20]. Similarly, the IL‐31 receptor α (IL‐31RA) expressed on sensory neurons is crucial in AD and cutaneous T‐cell lymphoma‐related itch [21]. IL‐17A, a key pathogenic cytokine in psoriasis, has been shown to act through its receptor IL‐17RA on sensory neurons, playing a regulatory role in skin injury healing [22]. However, the specific role of IL‐17RA, on sensory neurons in psoriasis‐related itch and inflammation remains to be fully elucidated.

Beyond their canonical role in transmitting peripheral signals to the central nervous system, sensory nerve fibers exhibit local regulatory capabilities through the release of neurotransmitters and neuropeptides, which modulate tissue‐resident immune responses [23, 24]. Functionally, sensory neurons are broadly categorized into peptidergic (PEPs) and nonpeptidergic (NPs) subtypes. While PEPs are classically defined by their secretion of neuropeptides such as substance P (SP) and calcitonin gene‐related peptide (CGRP), emerging evidence demonstrates that NPs also release immunomodulatory neuropeptides, including CGRP, chemokine‐like family member 4 (TAFA4), and somatostatin (SST), contributing to cutaneous immune homeostasis [25–27]. Recent investigations have elucidated the multifaceted roles of SP and CGRP in cutaneous pathophysiology. These neuropeptides interact with specific receptors on immune cells, thereby regulating their activation, proliferation, migration, and cytokine secretion, ultimately influencing immune responses in the skin [14]. Moreover, the sensory neurons are also the vital source of microRNAs (miRNAs) [28], ILs [29], and chemokines, which all play important roles in regulating the local immunity in the dermatosis [30]. This bidirectional neuro‐immune regulation is increasingly acknowledged as a critical mechanism in skin host defense, immune‐mediated inflammation, and tissue repair processes.

This review aims to examine the alterations in neural innervation within psoriatic skin and delineate the functional contributions of sensory neuron‐derived mediators—including ion channels, receptors, and neuropeptides—in modulating psoriasis‐associated immune dysregulation. By elucidating the role of neuro‐immune interactions in psoriasis pathogenesis, this review seeks to enhance our comprehension of the disease’s pathophysiology and offer novel perspectives for advancing research in psoriasis‐related neuro‐immune interactions.

## 2. Changes in Neural Innervation in Psoriatic Skin

Accumulating evidence demonstrates elevated neural innervation in psoriatic lesions compared to nonlesional skin, characterized by significant increases in nerve fiber number, density, total length, and intraepidermal nerve fiber counts [31–34]. Notably, these proliferating nerve fibers exhibit dense perineural networks surrounding immune cells and keratinocytes, suggesting their active involvement in psoriasis pathogenesis [35]. However, conflicting observations report reduced PGP9.5‐positive nerve fibers in severely inflamed psoriatic skin [36], and in some chronic psoriatic lesions, PGP9.5‐positive nerve fibers are absent [37]. These discrepancies may be due to differences in disease progression stages or methodological variations in histological assessment. A summary of these discrepancies is provided in Table 1.

**Table 1:** Summary of controversial findings and critical knowledge gaps in neuro‐dermatology of psoriasis.

Molecular target	Predominant finding	Contradictory finding	Probable explanation for discrepancies
PGP9.5+ nerve fiber density	Hyperinnervation: Multiple studies report significantly increased density, number, and length of PGP9.5+ fibers in psoriatic lesions compared to nonlesional skin [31–35].	Hypoinnervation/loss: PGP9.5+ fibers are reported to be reduced or absent in severely inflamed or chronic psoriatic plaques [36, 37].	These discrepancies may be due to differences in disease progression stages or methodological variations in histological assessment.
TRPA1 channel function	Pro‐inflammatory: TRPA1 is upregulated in lesions; Genetic deficiency (KO) or inhibition *attenuates* psoriasiform pathology and Th17 cytokine production [38].	Anti‐inflammatory: Genetic ablation or pharmacological inhibition of TRPA1 aggravates inflammation and pruritus, increasing levels of IL‐1β and TNF‐α [39].	Current studies mostly use global KO mice. TRPA1 is expressed on both sensory neurons (pro‐neurogenic inflammation) and nonneuronal cells (potentially protective), leading to mixed net effects.

**Figure 1:**
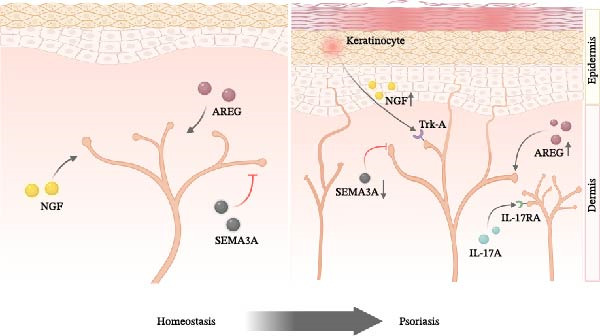
Neurotrophic mediators in psoriasis pathogenesis. NGF and AREG promote nerve fiber growth in psoriatic lesions via their receptors, while SEMA3A inhibits nerve growth. In psoriasis, elevated NGF and AREG and reduced SEMA3A expression drive epidermal hyperinnervation and inflammation. IL‐17A stimulates sensory neurons through IL‐17RA, enhancing nerve regeneration and inflammatory responses. (Created with BioRender, www.biorender.com). AREG, amphiregulin; IL‐17A, interleukin‐17A; IL‐17RA, interleukin‐17 receptor A; NGF, nerve growth factor; SEMA3A, semaphorin‐3A; Trk‐A, tropomyosin receptor kinase A.

The spatial patterning of cutaneous sensory innervation is governed by an intricate equilibrium between neurotrophic mediators and axonal repellent factors. Nerve growth factor (NGF) and amphiregulin exert potent neurotrophic effects [40, 41], whereas semaphorin‐3A serves as a key axonal guidance inhibitor [42]. Changes in nerve fiber density in psoriatic lesions may be regulated by these factors. Studies have shown reduced semaphorin‐3A expression alongside increased NGF and amphiregulin expression in psoriatic lesions [43, 44]. Meanwhile, increased NGF expression in keratinocytes has been identified as an early event in psoriasis pathogenesis, promoting nerve fiber growth by binding to the high‐affinity receptor tropomyosin receptor kinase A (Trk‐A) on epidermal nerve fibers (Figure 1) [45]. Notably, studies using IMQ‐induced mouse model revealed that NGF neutralization not only attenuated epidermal hyperinnervation but also ameliorated inflammatory pathology [46]. Moreover, sensory nerve denervation prior to the treatment of IMQ could significantly alleviate the induction of psoriasiform dermatitis [47], all of which establish a mechanistic link between neurotrophic signaling and cutaneous neuroimmunology in psoriasis pathogenesis. Additionally, IL‐17A, a well‐established key pathogenic factor in psoriasis, has been shown to directly stimulate sensory neurons through IL‐17 receptor A (IL‐17RA), promoting nerve regeneration in injured skin tissue [22]. However, whether IL‐17A alone acts as a neurotrophic factor in psoriasis or other inflammatory skin diseases remains to be further investigated.

Clinically, this pathological hyperinnervation correlates with neurosensory dysfunction manifesting as pruritus exacerbation and allodynia development. Quantitative analyses reveal positive correlations between intraepidermal nerve fiber density and subjective itch intensity scores in psoriatic patients [48]. Experimental models further illustrate that cutaneous denervation can mitigate scratch‐associated behaviors [46]. Additionally, elevated NGF levels directly modulate nociceptor sensitization, contributing to both pruriceptive and nociceptive dysfunction in psoriasis patients [49, 50]. Meanwhile, the semaphorin‐3A level is negatively correlated with itch intensity and severity of psoriasis [51].

In conclusion, the pathological hyperinnervation in psoriasis is predominantly driven by an imbalanced regulation of neurotrophic mediators. This abnormal increase in nerve fiber density directly contributes to clinical symptoms, including severe pruritus and allodynia. Moreover, this hyperinnervation establishes the anatomical foundation for enhanced neuro‐immune interactions.

## 3. Ion Channels and Receptors on Sensory Neurons in Psoriasis

Ion channels on sensory neurons serve as critical regulators of neuronal excitability through modulation of ion flux. The activation of ion channels on peripheral sensory neurons plays a crucial role in the transmission of itch and pain sensations and the development of local immune responses in psoriasis. The different roles of ion channels on sensory neuron in psoriasis are outlined in Table 2.

**Table 2:** Roles of ion channels on sensory neurons in psoriasis.

Ion channel	Location	Signal transduced	Ligands	Mode of action	Reference
TRPA1	• Sensory neurons • Keratinocytes • Dendritic cells	• Psoriasiform inflammation	Not specified directly	• Regulates dermal leukocyte infiltration and Th17 cytokine production • Effects remain controversial	[52, 53]
TRPV1	• Melanocytes • Fibroblasts • Vascular endothelium • Mast cells • Keratinocytes • Peptidergic C‐fibers	• Pruritus • Neurogenic inflammation	Thermal mechanical stress, bradykinin, prostaglandins	• Facilitates CGRP and SP release • Drives IL‐23 production in dendritic cells • Promotes inflammation	[38, 39, 54–59]
TRPV4	• Sensory neurons • Keratinocytes	• Itch • Inflammation	Heat, mechanical stress, arachidonic acid metabolites	• Regulates ATP expression • Antagonist alleviates psoriatic dermatitis	[60–62]
TRPM8	• Sensory neurons	• Cooling‐induced itch relief	Thymol	• Reduces dermal infiltration of neutrophils, dendritic cells, and Th17 lymphocytes	[63–66]
ASIC3	• Peripheral sensory ganglia	• Pruritus • Neurogenic inflammation	5‐HT, chloroquine, agmatine	• Promotes CGRP release • Aggravates inflammation via neurogenic pathway	[67–70]

### 3.1. TRP Channel Family

The TRP channel superfamily comprises 28 mammalian members, organized into six subfamilies (TRPC, TRPV, TRPM, TRPA, TRPP, and TRPML) that function as polymodal cation channels with preferential calcium permeability [52]. These molecular sensors regulate diverse physiological processes, including thermosensation, nociception, cellular homeostasis, and vascular tone [52]. TRP channels are predominantly located on sensory neurons and are key receptors for itch and pain sensations [53–55]. Increasing evidence suggests that TRP channels play a significant role in skin immune responses and inflammatory processes.

#### 3.1.1. TRPA1

Emerging evidence indicates marked upregulation of TRP ankyrin 1 (TRPA1) channel in both IMQ‐challenged murine psoriasiform models and human psoriatic epidermis [38]. TRPA1‐deficient mice demonstrate attenuated IMQ‐induced psoriasiform pathology, manifested by reduced dermal leukocyte infiltration, preserved epidermal barrier integrity, and suppressed Th17‐polarized cytokine production [38]. However, contrasting evidence emerges from independent investigations, revealing that both genetic ablation and pharmacological inhibition of TRPA1 paradoxically aggravate psoriatic inflammation and pruritus severity in preclinical models, with concomitant elevation of pro‐inflammatory mediators (IL‐1β, TNF‐α, and IL‐22) compared to wild‐type counterparts [39]. Notably, existing studies predominantly employ global TRPA1 knockout models. Given TRPA1’s pleiotropic expression across cutaneous cellular compartments—including keratinocytes, dendritic cells, and nociceptive neurons—its cell type‐specific functions remain mechanistically obscure. Future investigations should delineate the precise functional roles of TRPA1 in distinct cellular compartments and elucidate its mechanistic involvement in psoriatic pathogenesis, thereby facilitating the development of targeted therapeutic strategies. The conflicting roles of TRPA1 in different cellular compartments are critically evaluated in Table 2.

#### 3.1.2. TRPV1

The TRPV1, a polymodal cation channel integral to nociceptive signaling, transduces diverse stimuli including thermal, mechanical stress, and inflammatory mediators (e.g., bradykinin and prostaglandins) into calcium‐dependent cellular responses. Upon activation, TRPV1 undergoes a conformational change that facilitates extracellular Ca^2+^ influx. This rapid increase in intracellular calcium triggers the fusion of neuropeptide‐containing vesicles with the presynaptic membrane, leading to the exocytosis of SP and CGRP [56]. Cutaneous TRPV1 exhibits pan‐cellular expression patterns, with confirmed localization in melanocytes, fibroblasts, vascular endothelium, mast cells, keratinocytes, and PEP C‐fibers [57]. Emerging research has increasingly focused on elucidating the pathophysiological roles of TRPV1 in inflammatory dermatoses, encompassing psoriasis, AD, rosacea, and allergic contact dermatitis.

Studies have shown that TRPV1 is closely related to the occurrence and/or development of psoriasis. In IMQ‐induced psoriatic mice, TRPV1 knockout reduces the infiltration of CD45^+^ immune cells, mast cells, and CD3^+^ T cells, concomitant with reduced expression of inflammatory mediators (IL‐1β, IL‐6, IL‐23, and S100A8) [58]. Notably, TRPV1^+^ sensory fibers play a critical role in regulating psoriatic inflammation through neuroimmune interactions. Selective ablation of TRPV1^+^ sensory neurons impairs IL‐23 production in dermal dendritic cells, thereby suppressing IL‐23‐dependent γδT17 differentiation and subsequent inflammatory cell recruitment, ultimately ameliorating IMQ‐induced psoriasiform dermatitis [59]. Future research identified CGRP released upon TRPV1 activation binds to the RAMP1/CALCRL receptor complex on CD301b^+^ dDC. This ligand‐receptor interaction triggers downstream IL‐23 production [60]. The specialized pro‐resolving mediator resolvin D3 has been shown to modulate psoriatic inflammation through TRPV1‐mediated regulation of CGRP release from sensory neurons [61]. Additionally, systemic capsaicin treatment desensitizes TRPV1‐expressing sensory fibers and alleviates skin inflammation in psoriasiform mouse models [62], highlighting the therapeutic potential of TRPV1 modulation. TRPV1 has also been implicated in pruritus generation in psoriasis, with its expression levels positively correlating with itch severity [63]. These findings collectively establish TRPV1 as a critical mediator of both pruritus and inflammation in psoriasis, positioning it as a promising therapeutic target. Further investigation into the biological functions of TRPV1 and its mechanistic contributions to psoriatic pathogenesis may facilitate the development of targeted TRPV1‐based therapies for the treatment of psoriasis.

#### 3.1.3. TRPV4

TRPV4 channel functions as a multimodal sensor responsive to heat, mechanical stress, and lipid‐derived mediators such as arachidonic acid metabolites. Pathological analysis reveals significant TRPV4 upregulation in both human psoriatic plaques and IMQ‐induced murine psoriasiform lesions. TRPV4 knockout mice exhibit reduced skin inflammation with reduced expression of neuropeptides (CGRP, SP) and nerve fibers in psoriasiform dermatitis induced by IMQ treatment, and TRPV4 antagonists alleviate psoriatic dermatitis in mice [64]. Studies have confirmed that TRPV4 is highly expressed in dorsal root ganglion (DRG) tissues, and IMQ treatment further enhances its expression in neuronal tissues [65]. Importantly, TRPV4 on sensory neurons is involved in the chronic itch process in psoriasis, which has been identified as a mediator of pruritogenic signaling induced by miR‐203b‐3p, a keratinocyte‐derived miRNA implicated in psoriasis [66]. However, whether sensory neuron‐expressed TRPV4 also plays a critical role in regulating the inflammatory processes of psoriasis remains to be elucidated.

#### 3.1.4. TRPM8

TRPM8 could be directly excited by menthol and cold, which was also found to be crucial for cooling‐induced itch relief [67, 68]. Clinical investigations reveal positive correlations between TRPM8 expression levels and pruritus intensity scores in psoriatic patients [69]. Pharmacological activation of TRPM8 with thymol demonstrates therapeutic efficacy in murine psoriasiform models, ameliorating both pruritus and cutaneous pathology via calcium‐dependent signaling that suppresses dermal infiltration of neutrophils, dendritic cells, and Th17 lymphocytes [70]. These findings collectively establish sensory neuron‐expressed TRPM8 as a promising neuromodulator target for dual control of inflammatory and pruritic responses in psoriasis.

### 3.2. Acid‐Sensing Ion Channels (ASICs)

ASICs, members of the epithelial sodium channel/degenerin (ENaC/DEG) superfamily, function as proton‐activated cation channels with broad expression in both central and peripheral neural circuits. These channels critically regulate sensory transduction processes including mechanical sensing, chemical detection, and nociceptive processing [71]. The mammalian ASIC family comprises six molecularly distinct subtypes: ASIC1A, ASIC1B, ASIC2A, ASIC2B, ASIC3, and ASIC4, with ASIC1B and ASIC3 exhibiting predominant expression in peripheral sensory ganglia [65]. Mechanistic investigations demonstrate that ASIC3 activation by pruritogens (5‐HT, chloroquine, agmatine) drives itch signaling in murine models through direct neuronal excitation [72, 73]. Notably, genetic ablation of ASIC3 in sensory neurons attenuates psoriatic skin inflammation via impaired CGRP release, establishing a causal link between ASIC3‐mediated neurogenic inflammation and cutaneous immunopathology [74]. This highlights the role of ASIC3 in psoriatic neuroinflammation and suggests it as a potential therapeutic target.

### 3.3. Mas‐Related G‐Protein Coupled Receptors (MRGPRs)

The MRGPR family comprises a phylogenetically distinct subfamily of GPCRs, with over 50 murine members classified into subgroups A–H. In humans, this family encompasses eight characterized receptors: MRGPRX1‐X4, MRGPRD, MRGPRE, MRGPRF, and MRGPRG. Emerging studies delineate critical roles for specific MRGPR subtypes in modulating pruriceptive signaling, nociception, and neurogenic inflammation within sensory pathways.

While MRGPRs exhibit predominant expression in sensory neurons, mast cells and eosinophils have recently been identified as nonneuronal reservoirs of MRGPRX2 [75]. Functional characterization reveals that MRGPRC11 and MRGPRA3 colocalize with TRPV1^+^ nociceptors, mediating BAM8‐22‐evoked pruritus through histamine‐independent pathways [76]. MRGPRX1 has been implicated in neuronal activation and scratching behavior elicited by chloroquine and BAM8‐22 [77]. Notably, MRGPRX3 expression is significantly upregulated in lesional skin of patients with pruritic psoriasis, suggesting its potential involvement in disease pathogenesis [63]. Additionally, GPR15L, a pruritogenic mediator released by keratinocytes, exacerbates itch in psoriatic mice by targeting Mrgpra3 on sensory neurons [78]. Collectively, these findings underscore the pivotal role of MRGPRs in the regulation of chronic itch associated with psoriasis. However, the precise mechanisms underlying MRGPR‐mediated itch transmission and the distinct contributions of individual MRGPR subtypes to the progression of psoriatic skin inflammation remain incompletely understood and represent important areas for future investigation.

### 3.4. Oncostatin M Receptor (OSMR)

The OSMR can form heterodimeric receptors by binding to either IL‐31RA or gp130, thereby mediating downstream signaling upon activation by its ligands, IL‐31 or OSM. Studies have demonstrated that OSM expression is upregulated in various chronic pruritic skin diseases, including psoriasis, AD, and cutaneous T‐cell lymphoma. Selective knockout of OSMR in sensory neurons significantly reduces scratching behavior and alleviates epidermal thickening and other inflammatory manifestations in IMQ‐induced psoriatic mice [79]. These findings suggest that OSMR may participate in psoriatic skin inflammation by modulating sensory neuron function. However, it remains unclear whether the observed reduction in skin inflammation is directly attributable to decreased scratching behavior or results from the direct regulatory role of OSMR‐positive neurons in local inflammatory processes within psoriatic skin. Further investigation is required to elucidate the precise mechanisms underlying OSMR‐mediated regulation of psoriatic inflammation.

### 3.5. Protease‐Activated Receptor (PAR‐2)

The PAR family comprises four members, namely PAR‐1, PAR‐2, PAR‐3, and PAR‐4, which belong to the GPCR superfamily. Emerging evidence underscores the pivotal role of PAR‐2 in cutaneous neurogenic inflammation and inflammatory skin conditions, particularly AD [80]. Recent studies have further implicated PAR‐2 in the regulation of psoriasis‐related inflammation and pruritus. Notably, a marked upregulation of PAR‐2 expression has been observed in the lesional skin of patients with pruritic psoriasis [69], suggesting that PAR‐2 may mediate psoriatic itch transmission through neuroimmune modulation. Nonetheless, the precise regulatory mechanisms by which PAR‐2 contributes to the pathogenesis of psoriasis have yet to be fully elucidated.

### 3.6. Neurokinin‐1 Receptor (NK1R)

The NK1R, a member of the tachykinin receptor family, can be activated by multiple ligands, including SP, neurokinin A (NKA), neurokinin B (NKB), NPK, and neuropeptide‐γ (NKγ). NK1R is widely expressed in various cutaneous components, including immune cells, keratinocytes, and sensory nerve fiber terminals, playing a pivotal role in cutaneous itch transmission [81]. Clinical investigations have revealed a significant upregulation of both SP and NK1R expression in psoriatic lesions [63]. Phase II clinical trials have demonstrated that the NK1R antagonist serlopitant effectively alleviates pruritus in patients with mild to moderate psoriasis [82]. In IMQ‐induced psoriatic mouse models, stress stimulation was found to enhance the release of SP from sensory neurons, subsequently exacerbating cutaneous inflammation through NK1R activation. Notably, NK1R antagonists have been shown to partially reverse stress‐induced epidermal hyperplasia and SP elevation in these models [77]. These findings collectively establish the crucial role of NK1R in mediating psoriasis‐associated cutaneous inflammation and itch transmission.

### 3.7. IL17R

Previous studies have established that IL‐17A primarily acts on IL‐17R expressed on keratinocytes and immune cells, thereby exacerbating psoriatic inflammation—a key driver of the disease’s pathological mechanisms. The IL‐17 cytokine family encompasses six distinct ligands (designated IL‐17A through IL‐17F), among which IL‐17A functions as a primary mediator by activating downstream signaling cascades via its cognate heterodimeric receptor complex, composed of IL‐17RA and IL‐17RC subunits [83]. Recent evidence confirms that IL‐17RA and IL‐17RC are also present in DRG neurons [22, 84]. Notably, deletion of IL‐17RA and IL‐17RC from sensory neurons potently attenuates psoriasis‐like itch. This effect involves ERK pathway activation, a process mediated by ERK pathway‐dependent upregulation of TRPV4 in DRG neurons [85]. However, it remains undetermined whether IL‐17R on sensory neurons can be involved in the regulation of psoriasis inflammation through neuro‐immune pathway.

In summary, the diverse assortment of ion channels and specific receptors enables sensory neurons to serve as crucial “molecular integrators” within the psoriatic microenvironment. The aberrant sensitization and activation of these surface molecules on sensory neurons transduce immune signals in psoriatic skin into calcium‐dependent intracellular cascades. This mechanism actively perpetuates the inflammatory cycle by initiating neurogenic inflammation. Consequently, the pharmacological targeting of these upstream sensors offers a promising therapeutic strategy for the treatment of psoriasis.

## 4. Sensory Neuron‐Derived Mediators in Psoriasis

**Figure 2:**
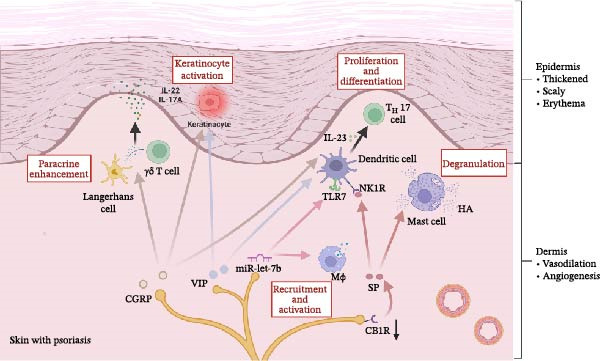
Sensory neuron‐derived mediators in psoriatic inflammation. Neuropeptides CGRP, SP, and VIP modulate immune responses in psoriatic skin. CGRP promotes IL‐23 production by dendritic cells, driving the IL‐23/Th17 axis and sustaining inflammation. SP, via NK1R, recruits immune cells and enhances cytokine production, worsening inflammation and pruritus. VIP promotes dendritic cell maturation and cytokine secretion. Sensory neuron‐derived miR‐let‐7b activates immune cells via TLR7, amplifying inflammation. (Created with BioRender, www.biorender.com). CB1R, cannabinoid receptor 1; CGRP, calcitonin gene–related peptide; γδ, gamma delta T cell; HA, histamine; IL‐17A, interleukin‐17A; IL‐22, interleukin‐22; IL‐23, interleukin‐23; Mø, macrophage; miR‐let‐7b, microRNA let‐7b; NK1R, neurokinin‐1 receptor; SP, substance P; Th17, T helper 17; TLR7, Toll‐like receptor 7; VIP, vasoactive intestinal peptide.

Emerging evidence has highlighted the crucial regulatory functions of neuropeptides derived from sensory nerve terminals in local cutaneous immune responses. Substantial research has demonstrated that specific neuropeptides, particularly CGRP and SP, can modulate the cutaneous immune microenvironment through interactions with immune cells, thereby influencing the initiation and progression of chronic inflammatory skin disorders (Figure 2). Furthermore, sensory neurons have been found to secrete miRNAs, which regulate local immune responses to influence psoriatic inflammation progression. Consequently, there has been growing scientific interest in elucidating the mechanistic roles of sensory neuron‐derived mediators in chronic inflammatory dermatoses, with particular emphasis on their involvement in psoriasis and related conditions.

### 4.1. CGRP

CGRP is a 37‐amino‐acid peptide that exists in two isoforms, CGRPα and CGRPβ, which are widely expressed in the central and peripheral nervous systems. In sensory ganglia, CGRP release is predominantly regulated through TRPV1 and TRPA1 cation channels, whose activation triggers calcium‐dependent exocytosis of CGRP‐containing vesicles [86].

Emerging evidence from recent studies has highlighted the pivotal role of CGRP in psoriasis pathogenesis. Histopathological analyses reveal striking increases in CGRP‐immunoreactive nerve fiber density within psoriatic plaques compared to normal skin [87], accompanied by elevated serum CGRP levels in psoriasis patients [88]. Intriguingly, expression profiling shows significant upregulation of canonical CGRP receptor components (RAMP1 and CALCRL) in lesional dendritic cells [89]. Preclinical studies using IMQ‐induced psoriasis models indicate that sensory neuron‐derived CGRP critically promotes DC‐dependent IL‐23 production via receptor‐mediated signaling, thereby initiating the IL‐23/Th17 inflammatory axis and sustaining cutaneous inflammation [59, 90]. Additionally, CGRP directly stimulates keratinocyte hyperproliferation by activating MAPK and NF‐κB signaling pathways, exacerbating psoriatic skin inflammation [91, 92]. In vitro mechanistic studies further demonstrated that CGRP enhances the release of IL‐17A and IL‐22 in Langerhans cell‐γδ T cell coculture systems through paracrine regulation, a process that amplifies psoriatic dermatitis symptoms [93].

Overall, these findings collectively demonstrate that CGRP significantly influences psoriatic inflammation through multifaceted interactions with various cutaneous cell types. Consequently, targeting CGRP and its associated signaling pathways represents a promising therapeutic strategy for psoriasis management.

### 4.2. SP

SP, an 11‐amino‐acid neuropeptide belonging to the tachykinin family, is widely distributed throughout the central and peripheral nervous systems [94]. This neuropeptide mediates cutaneous immune‐inflammatory responses through activation of neurokinin 1 receptor (NK1R) and MRGPR B2 (MrgprB2). SP exerts multiple pro‐inflammatory effects, including immune cell recruitment, cytokine modulation, and microvascular dilation.

Notably, immunohistochemical analyses have demonstrated significantly increased density of SP‐positive nerve fibers in psoriatic lesions compared to nonlesional skin, with predominant localization in the dermal papillary layers [95]. This anatomical distribution suggests a potential pathophysiological association with psoriatic features. Furthermore, SP‐NK1R interaction on DCs regulates DC maturation and activation, thereby promoting inflammatory cascades in psoriatic murine models [96, 97]. Recent investigations using IMQ‐induced psoriatic mouse models have revealed significant upregulation of SP expression in DRG tissues. Notably, deficiency of type I cannabinoid receptor (CB1R) on sensory neurons enhances SP release, leading to increased mast cell infiltration and keratinocyte proliferation in psoriatic skin, ultimately exacerbating cutaneous inflammation and itch‐related behaviors in these mice [98].

Moreover, SP serves as a key mediator in transmitting itch signals in psoriasis [99]. SP exacerbates pruritus by activating mast cells through MrgprB2 receptor, promoting their degranulation and subsequent release of histamine and other pruritogenic mediators [100]. Clinical evidence demonstrates elevated SP‐positive nerve and SP receptor expression in lesional skin of pruritic psoriasis patients, and the expression level of SP and its receptor was significantly correlated to pruritus intensity [101, 102], accompanied by increased MRGPRX2‐positive mast cell populations [103].

These findings collectively underscore the pivotal role of sensory neuron‐derived SP in modulating inflammation and pruritus in psoriasis, highlighting its significance in disease pathogenesis.

### 4.3. Vasoactive Intestinal Peptide (VIP)

VIP, a potent vasodilatory neuropeptide widely distributed in both the central and peripheral nervous systems, exhibits diverse biological effects. VIP primarily signals through the adenylate cyclase pathway and is involved in various physiological processes, including neurogenic inflammation, keratinocyte proliferation and differentiation, and angiogenesis [104]. VIP receptors are expressed on multiple cutaneous cell types, including keratinocytes, mast cells, and DCs.

Clinical investigations have revealed significantly elevated serum VIP levels in psoriasis patients compared to healthy controls, with a marked increase in VIP‐positive nerve fibers in psoriatic lesions relative to both normal skin and control skin [105]. In vitro studies have demonstrated that VIP promotes DC maturation, subsequently inducing CD4^+^ T cell proliferation [106]. Moreover, VIP also could induce the production of IL‐6 and IL‐8 from keratinocytes, thereby contributing to the development of inflammatory dermatoses such as psoriasis [107]. However, the precise mechanistic role of VIP in psoriasis pathogenesis remains incompletely understood, warranting further investigation to elucidate its contribution to disease progression.

### 4.4. miRNAs

miRNAs, small noncoding RNAs of 20–24 nucleotides (nt), typically regulate gene expression by binding to the 3^′^‐untranslated region (3^′^‐UTR) of target mRNAs to suppress translation or promote mRNA degradation. Emerging evidence reveals a noncanonical role wherein miRNAs function as extracellular signaling mediators that facilitate intercellular communication. Notably, miRNAs serve as neural signaling mediators critically involved in transmitting pruritic and nociceptive stimuli [108, 109].

Studies have shown that sensory neuron‐derived miRNAs further contribute to local immune regulation. For instance, miR‐let‐7b – an endogenous ligand for toll‐like receptor 7 (TLR7)—activates dermal dendritic cells through TLR7 signaling, thereby promoting IL‐23 secretion and exacerbating psoriatic pathology [110]. Furthermore, chronic stress exposure could upregulate miR‐let‐7b release from sensory neurons, enhancing macrophage recruitment and activation in the skin, which ultimately accelerates psoriasis progression [28]. Collectively, these findings demonstrate that sensory neurons modulate cutaneous immune processes not only via neuropeptide secretion but also through the release of additional mediators such as miRNAs. However, current research has only characterized regulatory functions of a limited miRNA subset in psoriasis pathogenesis. Future investigations should delineate the roles of other sensory neuron‐derived miRNAs in immune regulation across psoriasis and other inflammatory dermatoses.

In summary, the release of bioactive mediators constitutes the primary effector function of sensory neurons within the psoriatic microenvironment. These mediators function as a critical interface between the nervous and immune systems. The findings highlight that sensory neurons are active contributors to cutaneous pathology, rather than passive signal transducers. Therefore, inhibiting the release or receptor binding of these specific mediators presents a targeted therapeutic approach to disrupt the neuro‐immune circuitry underlying psoriasis.

## 5. Conclusion

**Figure 3:**
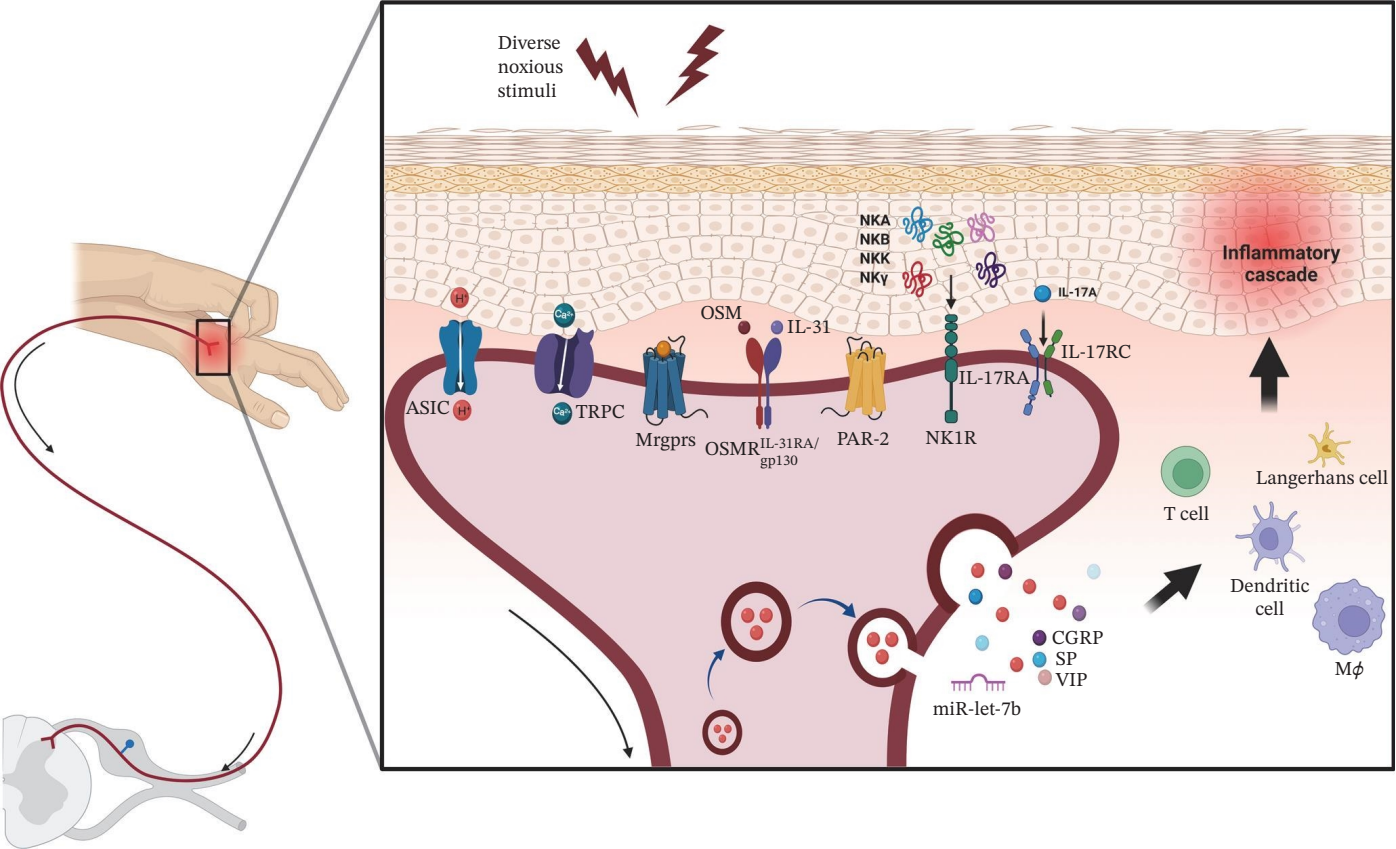
Sensory neuron receptors and mediators in psoriasis neuro‐immune regulation schematic of sensory neuron receptors and mediators driving neuro‐immune interactions in psoriasis. (Created with BioRender, www.biorender.com). ASIC, acid‐sensing ion channel; CGRP, calcitonin gene–related peptide; gp130, glycoprotein 130; IL‐17A, interleukin‐17A; IL‐17RA, interleukin‐17 receptor A; IL‐17RC, interleukin‐17 receptor C; IL‐31, interleukin‐31; IL‐31RA, interleukin‐31 receptor A; miR‐let‐7b, microRNA let‐7b; Mø, macrophage; MRGPRs, Mas‐related G protein–coupled receptors; NKA, neurokinin A; NKB, neurokinin B; NKγ, neurokinin gamma; NK1R, neurokinin‐1 receptor; NKK, neurokinin K; OSM, oncostatin M; OSMR, oncostatin M receptor; PAR‐2, protease‐activated receptor 2; SP, substance P; TRP, transient receptor potential channel; VIP, vasoactive intestinal peptide.

This review proposes an integrated neuro‐immune framework to elucidate the complex role of peripheral sensory neurons in psoriasis, moving beyond the traditional view of immune‐keratinocyte crosstalk. By systematically synthesizing current evidence, we highlight three critical dimensions of neurogenic inflammation: (1) the aberrant hyperinnervation patterns in psoriatic lesions, (2) the mechanistic roles of specific ion channels, and (3) the immunomodulatory effects of neuron‐derived mediators. Extensive research has elucidated the functional significance of specific receptors expressed on these neurons and the neuropeptides they secrete in driving psoriatic inflammation. These neuropeptides, in turn, critically influence the physiological functions of skin‐resident and infiltrating immune cells, thereby regulating local immune responses in psoriasis and other dermatoses (Figure 3). Among the neuropeptides implicated, CGRP and SP have been the most extensively characterized. However, the precise mechanisms and functional contributions of other neuropeptides, such as PACAP, SST, and NPY, in psoriasis pathogenesis remain poorly defined and constitute a significant knowledge gap requiring further investigation.

Recent advances have significantly expanded our understanding, revealing that sensory neuron function not only as neuropeptide secretors but also as direct sources of key inflammatory cytokines. For instance, neuronal secretion of IL‐6 has been demonstrated to modulate regulatory T cell (Treg) differentiation, thereby influencing peripheral immune processes [102]. This discovery underscores the broader cytokine secretion capacity of sensory neurons in peripheral immune regulation.

A critical unresolved question is whether activated sensory neurons in psoriatic skin modulate disease progression exclusively through neuropeptide/cytokine release, or if additional mechanisms (e.g., direct neuron‐immune cell synaptic communication, modulation of vascular or epithelial function) are involved. Moreover, although the increased density of CGRP‐ and SP‐positive nerve fibers within psoriatic lesions is well‐documented, the causal relationship between this neuroplasticity and specific alterations in the cutaneous immune microenvironment remains elusive.

Critically, we emphasize that these neural pathways are not merely pathophysiological bystanders but viable therapeutic targets. Translating these mechanistic insights into clinical practice represents the next frontier in psoriasis management. Specifically, TRPV1 antagonists hold promise as a treatment for psoriasis by dampening the upstream initiation of neurogenic inflammation and itch signaling at the nerve terminal [111]. Furthermore, NK1R blockers (e.g., serlopitant) have demonstrated potential efficacy in mitigating psoriatic pruritus by interrupting the SP‐mast cell axis [82]. It is noteworthy that rimegepant, a CGRP inhibitor, has been approved for the treatment of migraines. Reports indicate that it significantly improves cystic acne in clinical use [112]. In addition, the use of erenumab, an anti–CGRP receptor monoclonal antibody, also improve the erythema and flushing associated with rosacea [113], while direct clinical validation in psoriasis is currently lacking. Concurrently, emerging interdisciplinary strategies are expanding the therapeutic landscape beyond traditional pharmacotherapy. For instance, novel biomaterial‐based platforms, such as spatially confined nanozyme hydrogels combined with nitric oxide gas therapy, have demonstrated enhanced efficacy in treating psoriasis by synergizing anti‐inflammatory effects with tissue repair mechanisms [114]. These advancements highlight the potential of combining neuro‐immune modulation with advanced delivery systems to achieve superior clinical outcomes.

Future research directions should focus on elucidating the aberrant mechanisms underlying neuro‐immune interactions in psoriatic skin. Such investigations could provide innovative and targeted therapeutic strategies for psoriasis management, potentially advancing the field of psoriasis treatment and improving clinical outcomes.

## Funding

This work was supported by the National Natural Science Foundation of China (Grant 82573979).

## Ethics Statement

The authors have nothing to report.

## Consent

The authors have nothing to report.

## Conflicts of Interest

The authors declare no conflicts of interest.

## Data Availability

Data sharing is not applicable to this article as no datasets were generated or analyzed during the current study.
